# Impact of Cash for Health Assistance on Healthcare Access and Health-Seeking Behaviors for Families of Pregnant Women in Sindh, Pakistan

**DOI:** 10.3390/ijerph22121843

**Published:** 2025-12-10

**Authors:** Faiza Rab, Ahmad Wehbi, Asma Hasnat, Chelvi Singeswaran, Mohamed Aliyar Ifftikar, Salim Sohani

**Affiliations:** 1 Canadian Red Cross, Ottawa, ON K2P 2N1, Canada; ahmad.wehbi@redcross.ca (A.W.); chelvi.singeswaran@redcross.ca (C.S.); salim.sohani@redcross.ca (S.S.); 2 Norwegian Red Cross, 0186 Oslo, Norway; asma.hasnat@redcross.no (A.H.); mohamed.aliyar.iffthikar@redcross.no (M.A.I.)

**Keywords:** cash support, multipurpose cash assistance, humanitarian response, maternal health, child health, nutrition

## Abstract

Background: The 2022 Pakistan floods devastated healthcare access for pregnant women in already impoverished areas in Sindh province. This study examines how Cash for Health assistance (CH) of USD 112 alleviated financial burdens and improved maternal health outcomes and resilience, bridging a critical literature gap on cash effectiveness in humanitarian crises. Methodology: This study used a mixed-methods approach to assess the CH assistance intervention for families of pregnant/lactating women in flood-affected rural Sindh, Pakistan. A pre-post quantitative analysis of baseline (May–June 2024) and endline (August–November 2024) survey data in ~100 villages (Jamshoro/Sehwan) examined changes in healthcare access, expenditure, and preferences using *t*-tests, proportion tests, and multivariable regression. Concurrently, five qualitative case studies from key informant interviews provided thematic content analysis, triangulating findings on economic, health, and social impacts. Results: Respondents predominantly had low literacy rates and were from households of daily wage laborers in vulnerable, flood-affected areas. While income and education remained low, instances of forgone care due to financial barriers increased (68% to 97%, *p* < 0.001). CH significantly improved healthcare access (58% to 98%, *p* < 0.001). Access to regular physicians (20% to 69%) and private facilities (10% to 41%) notably expanded. Healthcare expenditure significantly increased from USD 9.3 to USD 25, with a shift in spending preference towards medication, consultations, and diagnostics. CH also significantly improved food security (21% to 97%), meal frequency, and overall household stability, including reducing domestic violence. Qualitative data emphasized pre-existing vulnerabilities and CH’s role in addressing health, nutrition, and psychosocial needs. Conclusions: CH significantly improved healthcare access and reduced financial burdens for vulnerable pregnant women post-disaster. However, a sustainable impact requires integrated “cash plus” models, combining financial aid with stronger health systems, psychosocial support, and literacy for long-term resilience.

## 1. Background

Pakistan’s geography and climate make it highly vulnerable to natural disasters, particularly to severe and recurring floods. As millions in Punjab and Khyber Pakhtunkhwa struggle through the devastation of the 2025 monsoon floods, the country is forced to relive the trauma of 2022, when climate change drove one of the worst flood disasters in its history, particularly devastating Sindh province [1,2,3]. The province of Sindh bore the brunt of the disaster, with 1600 fatalities and two million homes destroyed. The widespread damage to infrastructure and essential services severely hindered access to critical healthcare. Notably, the regions most severely impacted by the floods were already characterized by pre-existing, high levels of poverty, rendering the affected populations exceptionally vulnerable to the disaster’s multi-faceted consequences [4,5]. This pervasive impoverishment meant that many families lacked the financial resilience to cope with the sudden loss of homes, livelihoods, and access to basic services, deepening their reliance on external aid for survival and recovery.

The United Nations Population Fund (UNFPA) estimated that 650,000 pregnant women resided in flood-affected areas, with approximately 73,000 deliveries anticipated in the immediate aftermath [6,7]. The floods decimated over 1000 health facilities in Sindh, preventing women from accessing essential prenatal care, safe delivery services, and skilled birth attendants [6,8]. This further strained Pakistan’s already fragile healthcare system [6]. Overcrowded relief camps, coupled with inadequate access to clean water and food, led to increased rates of malnutrition, the spread of waterborne diseases, and elevated risks of pregnancy complications such as anemia and preterm labor [7,8,9]. Poor living conditions in these camps also created significant challenges for women regarding basic hygiene, privacy, and access to clean sanitation facilities [10].

Socio-cultural barriers compounded these difficulties, with an insufficient number of female healthcare workers and a lack of private spaces in temporary shelters hindering women’s access to essential reproductive health services [8,10]. Moreover, the absence of secure areas in displacement zones escalated the risk of gender-based violence, including sexual harassment, abuse, exploitation, and increased rates of child and forced marriages [9]. The displacement, loss of livelihoods, and pervasive uncertainty about the future also inflicted severe psychological distress on women [9].

In response, humanitarian organizations distributed clean delivery kits, newborn baby kits, and dignity kits and health services via mobile health units [6]. However, two years after the floods, the affected communities continued to grapple with their aftermath. Recognizing the significant financial barriers to healthcare access, which were exacerbated by the pre-existing poverty within the affected population, the Norwegian Red Cross (NorCross), in collaboration with the Pakistan Red Crescent Society (PRCS), initiated Multi-Purpose Cash Assistance for Health (CH) alongside basic health education for pregnant women in Sindh. This support, providing eligible families with approximately USD 112 USD (In total, approximately PKR 32,000 was distributed, which in mid 2024 comes out to be approximately USD 130. Resource: https://www.oanda.com/currency-converter/en/?from=PKR&to=USD&amount=36000, accessed on 8 September 2025) in installments, aimed to alleviate financial burdens and facilitate access to vital maternal healthcare services and other urgent needs.

While cash and voucher assistance (CVA) has gained significant traction in humanitarian responses in recent years, becoming an increasingly central component of emergency aid efforts worldwide, its widespread adoption as a primary modality is relatively novel. There remains a notable gap in the literature regarding its comprehensive use and medium to long-term impact, particularly in complex and protracted emergencies, and its specific effectiveness in addressing the multifaceted vulnerabilities of women and pregnant women in disaster contexts [11,12]. This study contributes to bridging this gap by examining the application and outcomes of CH in supporting maternal health during post-flood recovery.

Given that cash and voucher assistance (CVA) as a primary humanitarian modality is relatively novel, with recognized gaps in the existing literature regarding its comprehensive use and impact in complex emergency settings, this study seeks to contribute crucial empirical insights. This research aimed to provide an evidence-based examination of the CH assistance program implemented in Sindh, Pakistan. The primary focus is to understand its effects on healthcare services for pregnant women and their families in the context of natural disasters. The objectives of this study were as follows:

1. Explore the impact of CH on improving access to essential healthcare services for families of pregnant women within the post-flood recovery phase.
2. Examine whether the CH assistance reduced the financial burden on families of pregnant women affected by the disaster.
3. Assess whether the factors associated with healthcare expenditure (specifically access to healthcare services and preference for healthcare spending) changed between the baseline and endline periods following the Cash for Health intervention.

## 2. Methodology

A mixed-methods approach was used to assess the CH assistance program in rural Sindh, Pakistan, focusing on its impact on healthcare access and financial well-being for pregnant women and their families following the 2022 monsoon floods. A total of up to approximately USD 112 was disbursed to eligible families in multiple installments. Non-randomized sampling was used based on eligibility criteria. Eligible participants for the CH intervention included families of pregnant or lactating women with a mid-upper arm circumference (MUAC) under 23 cm, a history of past pregnancy complications with low-birth-weight babies, or those whose livelihoods were affected by the floods or other disasters.

The quantitative component employed a repeated cross-sectional design, analyzing secondary data from baseline (May–June quantitative component 2024) and endline (August–November 2024) surveys conducted among pregnant or lactating women from families benefiting from the CH intervention across over 100 villages in Jamshoro and Sehwan Talukas in Sindh province. These surveys, encompassing demographic, socioeconomic, health expenditure, preferences, and access questions, provided the quantitative foundation.

Descriptive data analysis involved calculating mean values and 95% confidence intervals for continuous variables and frequency distributions for categorical and binary variables. Furthermore, changes from baseline to endline were measured as differences using independent two sample *t*-tests for continuous variables and two sample proportion tests for categorical and binary variables.

Finally, the study utilized a multiple regression approach specifically designed for the repeated cross-sectional (RCS) nature of the data. Given that the baseline and endline samples consist of independent, non-matched individuals, pooled regression models were not appropriate. Therefore, two separate regression models were estimated: one for the baseline sample and one for the endline sample. This strategy serves to address the refined Research Objective 3: to determine if the predictive structure of healthcare expenditure changed following the intervention. By comparing the magnitude and statistical significance of the key predictor coefficients between the baseline and endline models, we examined whether the impact of certain factors (e.g., financial/preference barriers) in predicting total expenditure shifted after the Cash for Health transfer.

The dependent outcome variable for both models was Healthcare Expenditure (USD). The independent predictor variables (covariates) were selected based on theoretical relevance to the intervention’s mechanism, focusing on access and preference indicators. Covariates present in the final regression model were omitted only if they exhibited high collinearity with retained variables or showed no statistically significant association with the dependent variable (Healthcare Expenditure) in preliminary analyses, ensuring model parsimony and stable coefficient estimates. All quantitative analysis was performed using Microsoft Excel Version 2510 [13] and Stata version 14 [14].

The qualitative component involved a content analysis of insights derived from five case studies, compiled from key informant interviews conducted by the NorCross and PRCS teams with pregnant or lactating women in the community. These case studies, serving as secondary data, explored thematic areas including economic hardship and food insecurity, maternal and child health problems, disaster impact, domestic abuse and violence, and empowerment. We used a manifest content analysis approach, with two independent reviewers extracting relevant data, grouping codes into sub-themes, and connecting them to the main study objectives. Discrepancies between reviewers were resolved through consensus, and key quotes were extracted to substantiate the findings.

Ethical considerations: This study, an internal quality assurance initiative by the Pakistan Red Crescent Society, aimed at improving the health and well-being of pregnant women and families affected by floods. This falls under routine organizational monitoring and evaluation, and a formal ethics review was not required by institutional guidelines. Stringent ethical principles guided the research, which used only anonymous, de-identified secondary data from reports previously published by NorCross. Consent for the use of these data for publication and dissemination was provided by the Pakistan Red Crescent Society. This approach eliminated direct interaction with individuals and ensured participant privacy and confidentiality.

## 3. Results

The average age of respondents at endline was 28 years, significantly lower compared to baseline at 31 years (*p* < 0.001), Table 1. Household income, reported only at baseline, averaged USD 54 per month, with 81.8% of primary earners working as daily wage laborers. Most households consistently had one child under 2 years old at both baseline (74.9%) and endline (81.1%), followed by households with no children under age 2; however, the difference between baseline and endline was significant. Educational attainment remained low, with 87% of participants reporting no formal education throughout the study (Table 1). A significant increase in the reporting of barriers to accessing health services, identified as limited availability of services, financial constraints, and transportation challenges, was observed at endline compared to baseline among participants. Similarly at baseline, 68% of respondents reported having previously forgone medical care due to financial constraints, with this figure increasing to 97% at endline (*p* < 0.001), see Table 1.

Participants allocated an average of 22% of the total cash received towards healthcare needs. Table 1 also shows that nearly half of the participants (47%) received four installments of the CH assistance, while 31% received two installments.

Overall, participants’ ability to access healthcare services saw significant improvements, all across (*p* < 0.001) baseline to endline, with the proportion of respondents reporting access rising from 58% to 98%. While access to regular physicians and private facilities notably expanded, increasing from 20% to 69% and 10% to 41%, respectively, access to emergency services decreased, moving from 71% at baseline to 30% at endline. Access to government facilities also increased, from 43% to 58%.

The healthcare expenditure also significantly increased from an average of USD 9.3 spent in the last 3 months at baseline to USD 25 at endline. Regarding healthcare spending preferences, there was a consistent shift towards prioritizing core medical needs. Preference for medication spending saw a dramatic rise from 30% to 95%, and for consultations, it moved from 6% to 71%, *p* < 0.001. Preferences for treatment and laboratory testing also increased significantly, reaching 44% and 40%, respectively, at endline, up from 10% and 5%, respectively, at baseline. Spending preference for labor and delivery remained unchanged and very low, at 0.4% across both periods, and was not statistically significant.

The variables in this table are grouped solely by conceptual similarity (e.g., all Financial Burden indicators, all Access indicators) to facilitate a clear descriptive overview of the sample and the measured pre-post changes. In this pre-post analysis, all variables related to Access, Financial Burden, and Expenditure are considered primary outcomes of the Cash for Health intervention.

Beyond healthcare access, a significant finding regarding spending patterns was the substantial increase in easy access to food following the CH intervention. This access rose sharply from 21% at baseline to 97% at endline (*p* < 0.001). Correspondingly, a significant change in meal frequency was observed. At baseline, a significantly higher percentage of respondents reported consuming two meals per day; this significantly shifted to a higher number of participants reporting access to three meals per day at endline (*p* < 0.001), Table 1. Furthermore, Table 1 illustrates that when participants were asked to choose between healthcare and other essential needs, there was a significant increase at endline in preference for food, livestock, and rent. Concurrently, there was also a significant increase at endline in borrowing money, asking for support from the family, and selling livestock.

Table 2 presents the outcomes of the multiple regression analysis examining healthcare expenditures. At baseline, the sole factor with a statistically significant association with healthcare expenditure was a household’s stated preference for consultations with healthcare providers. Households indicated that at baseline, the families of pregnant women who preferred to have consultations spent, on average, PKR 3595 (USD 12.7) more on healthcare than those who did not have that preference (*p* = 0.001), after accounting for sociodemographic and other pertinent variables.

By endline, we observed other additional significant associations with healthcare spending. First, individuals with no formal education spent PKR 4120 (USD 14.5) less in healthcare expenses compared to those with some level of education (*p* = 0.003). Second, those who utilized government healthcare facilities spent PKR 4568 PKR (USD 16.1) less than individuals who did not, *p* < 0.01. Moreover, endline data revealed significantly higher healthcare expenditure correlating with stated preferences for consultations (USD 12.7), treatment (USD 10.8), laboratory diagnostics (USD 10.9), and labor and delivery (USD 27.1). The only exception was a preference for medications, where no significant association was observed (see Table 2).

**Figure 1:**
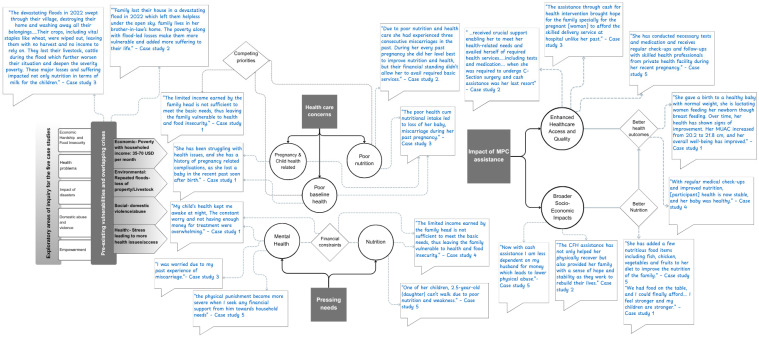
Illustration of qualitative findings.

Our qualitative analysis reveals critical insights into the multifaceted challenges faced by highly vulnerable, low-income households in the aftermath of devastating flooding events, and the transformative impact of cash assistance on their health and well-being. Five in-depth case studies, focusing on pregnant women, highlight severe pre-existing vulnerabilities and the significant improvements facilitated by direct financial aid. An illustration of the thematic underpinnings, identified themes, sub-themes, and quotations is presented in Figure 1, and details are provided here:

### 3.1. Pre-Existing Vulnerabilities and Overlapping Crises

All five analyzed households were characterized by very low income, with reported monthly earnings between PKR 10,000 and 20,000 (USD 35 to 70) per month, classifying them as extremely impoverished. The recurrent flooding events of 2022 exacerbated their vulnerability, leading to substantial losses of homes, belongings, crops, and livestock. One family, for instance, lost their home in a devastating 2022 flood, leaving them helpless and forcing them into precarious living situations. Another family lost their livestock and cattle during the flood, further worsening their situation and deepening their poverty, highlighting the cascading economic impacts. In three cases, repeated flood exposure significantly heightened household vulnerability. Beyond environmental disasters, pre-existing economic stress was compounded by severe social issues in one household, where domestic violence and physical abuse were linked to the spouse’s untreated mental health issues.

### 3.2. Identified Healthcare Concerns

A pervasive theme across all case studies was the women’s inability to address critical *pregnancy and child health needs* due to severe financial constraints. The limited income earned by the family head was insufficient to meet basic needs, leaving families vulnerable to health and food insecurity. A concerning finding was the high incidence of adverse pregnancy outcomes, with four of the five participants reporting a history of stillbirths and miscarriages. *Poor health and nutritional intake* often led to these tragic losses during past pregnancies.

Participants also reported *poor baseline health status*, stemming from chronic malnutrition and the prevalence of both chronic and infectious diseases. One participant attributed three consecutive miscarriages in the past to poor nutrition and healthcare. Children, too, frequently suffered from illnesses like fever and pneumonia, necessitating constant medical attention. Consequently, *access to medical consultations*, diagnostic tests, and essential medications emerged as a critical, yet largely unmet, need due to significant financial barriers. The poor financial standing simply did not allow them to access health services.

#### 3.2.1. Pressing Needs

*Nutrition* was universally identified as a primary concern, with all women reporting that inadequate dietary intake negatively affected their own health and that of their children. The dire consequences of this nutritional deficit were evident, as one child, at two and a half years old, could not walk due to poor nutrition and weakness.

Beyond physical health, the qualitative data subtly indicated an underlying *need for mental health support.* Although not explicitly stated, at least three participants indirectly referenced the significant impact of stress and worry on their psychological well-being. The constant worry about a child’s health and not having enough money for treatment was overwhelming. In one particularly distressing case, the spouse’s mild mental health disorder manifested as ongoing domestic violence, with the woman enduring frequent physical abuse and a profound sense of helplessness and lack of support. The physical punishment became more severe when she sought financial support for household needs.

#### 3.2.2. Impact of Cash Assistance

The provision of cash assistance demonstrably improved health outcomes and overall well-being across all participating households, addressing both immediate and underlying vulnerabilities.

*Enhanced Healthcare Access and Quality:* A critical finding was that all participants reported improved access to health services following the receipt of cash assistance. This included the ability to afford essential medications, medical consultations, diagnostic tests, and crucial delivery care. One participant received crucial support enabling her to meet her health-related needs and avail herself of required health services, including tests and medication, particularly when she needed a C-section and cash assistance was her last resort.

Notably, all participants were able to access private healthcare services, which they uniformly perceived as offering higher quality due to their greater cost. Pregnant women, in particular, emphasized that cash assistance enabled them to seek skilled delivery services at private hospitals, thereby improving the overall quality of maternal healthcare received. This assistance brought hope for the family, especially for pregnant women, to afford skilled delivery services at hospitals, unlike in the past. This enhanced access to quality care contributed to significant improvements in health status, particularly for mothers and children. All participants reported a general enhancement in the overall well-being of their households, and three participants observed improvements in their mid-upper arm circumference (MUAC) readings, with some increasing by 1.5–2 cm and even exceeding 23 cm, indicating improved nutritional status. One participant gave birth to a healthy baby with normal weight and, as a lactating woman, was breast-feeding; her health showed signs of improvement, with her MUAC increasing from 20.2 to 21.8 cm, and her overall well-being improved.

*Broader Socio-Economic Impacts:* Beyond healthcare, cash assistance had a significant positive impact on other identified needs, particularly *nutrition*. All participants highlighted the direct link between financial resources and improved dietary intake, enabling them to purchase more nutritious food items for their families. One participant added nutritious food items like fish, chicken, vegetables, and fruits to her family’s diet to improve their nutrition. Families reported having food on the table and being able to afford necessary items, feeling stronger, and seeing their children become stronger.

Crucially, the cash assistance fostered a sense *of hope and stability*, empowering families to begin rebuilding their lives after the floods. The assistance not only helped these families physically recover but also provided them with a sense of hope and stability as they worked to rebuild their lives.

Perhaps one of the most profound impacts was the empowerment of women to *break cycles of abuse,* particularly in cases of domestic violence. With cash assistance, women reported being less dependent on their husbands for money, which led to lower physical abuse.

## 4. Discussion

Despite global efforts, the financial burden of healthcare, food insecurity, and limited access to essential services remain critical challenges for vulnerable populations, particularly pregnant women in crisis-affected regions [15,16,17]. This study sought to understand how CH assistance directly addressed these issues in flood-affected Sindh, Pakistan, by examining its impact on healthcare access, expenditures, and household financial resilience, ultimately revealing its potential to transform health-seeking behaviors in resource-constrained, disaster-prone settings.

The study population, consistently vulnerable due to low education and reliance on daily wages, faced significant hardships amplified by the presence of younger children and a shift towards even younger respondents at endline. Case studies vividly demonstrated the challenges for pregnant and lactating women in flood-affected, low-income households in Sindh, where extreme poverty and recurrent climate shocks severely disrupted livelihoods, housing, and food security. These systemic issues directly compromised maternal and child health, leading to pregnancy complications, stillbirths, and chronic undernutrition. Ultimately, our findings underscore the potential of CH assistance to enhance health outcomes and fortify economic resilience in at-risk households, especially immediately after an emergency.

### 4.1. CH’s Impact on Healthcare Access and Expenditure

The CH assistance program in Sindh significantly improved healthcare access and physical health for flood-affected pregnant women. Notably, this occurred even though participants reported spending only a quarter of the CH on direct healthcare needs. While baseline spending was linked to preferences for consultations, endline data revealed an equity gap, with lower expenditures associated with a lack of formal education and reliance on government facilities. Conversely, broader preferences for healthcare services correlated with higher spending, suggesting that financial empowerment led to more comprehensive health investments [18]. This compellingly indicates that financial capacity is a key determinant of healthcare access for this vulnerable population.

The intervention also spurred a positive shift in health-seeking behaviors. Beneficiaries transitioned from primarily seeking emergency-only care to an increase in regular and preventive healthcare visits. Access to both government and private facilities improved, particularly for private procedures like C-sections, pointing to enhanced quality of care for pregnant women and their families. This aligns with recent findings that private hospitals generally yield higher patient satisfaction than public facilities [19]. Interestingly, despite improved access, participants reported more barriers like limited availability, cost, and transportation at endline. This paradox likely reflects increased health awareness and care-seeking attempts, leading to a greater recognition of unmet needs rather than an actual deterioration in access. Similarly, more participants reported forgoing care due to financial constraints post-intervention, possibly indicating heightened health literacy and more frequent attempts to seek care, thus encountering barriers when funds or services were insufficient. Health literacy is crucial for individuals to understand health information, develop skills, and gain autonomy over personal health decisions [20].

### 4.2. CH’s Role in Reducing Financial Burden

Financial constraints consistently emerged as the primary barrier to timely and appropriate healthcare access, especially for vulnerable populations [21,22]. Women consistently reported being unable to afford medical consultations, essential medications, diagnostic tests, and skilled delivery care. These financial challenges were often compounded by underlying chronic illnesses, poor nutrition, and, in some cases, psychosocial stress or domestic violence. The significant mental toll of financial worry and prior traumatic experiences suggests an unmet need for mental health support. Similarly to our findings, Dawkins et al. [22] identified additional barriers such as delays, sociocultural perceptions, empowerment issues (e.g., lack of decision-making power), information gaps, low perceived needs, lack of treatment acceptability, and insufficient healthcare providers and transportation.

Beyond immediate health impacts, the cash assistance fostered broader household stability. Women described a renewed sense of control over their lives, with some participants noting a reduction in domestic violence due to decreased financial dependence. CH facilitated not only physical recovery but also emotional resilience, offering families the opportunity to begin rebuilding their lives after the trauma of the floods. This aligns with evidence that financial inclusion empowers women by improving access to education, healthcare, and savings, while increasing their role in household and community decision-making [23,24,25,26]. However, it is crucial to acknowledge that while cash incentives offer benefits, there is a need for caution regarding potential unintended consequences, such as targeting and isolation issues, and challenges with sustainability and building long-term resilience [27].

### 4.3. Broader Impact: Food Security and Long-Term Needs

A major highlight of the intervention’s impact was the marked improvement in food security and dietary intake. The percentage of households reporting access to nutritious food rose dramatically, and daily meal frequency also increased, suggesting that CH provided critical support in meeting immediate nutritional needs and aligning with participants’ increased preference for food at endline. The CALP Network’s 2019 report, featuring the Nobo Jatra program, similarly highlighted how cash transfers served as a nutritional safety net, with recipients prioritizing spending on food, directly contributing to improved health and nutrition outcomes for mothers and children [12].

Despite improved access to nutritious food, a higher percentage of participants reported skipping meals, and more households relied on borrowing and family support—even with cash assistance. This may reflect the decline in external emergency aid over time, which typically tapers off post-disaster [28]. As a result, CH alone may not be sufficient to meet the evolving and ongoing needs of affected families, underscoring the importance of integrated, long-term support strategies.

### 4.4. Recommendations

While the CH intervention significantly improved healthcare access for flood-affected pregnant women, the study identified critical gaps requiring further attention to achieve sustainable impact. Despite improvements, many households continued relying on negative financial coping strategies, like borrowing and skipping meals, indicating the need for complementary and sustained economic interventions. Although government health facilities were more frequently accessed at endline, their capacity for comprehensive maternal care raises concerns about long-term affordability and continuity, especially given the temporary access to higher-quality private care during the cash assistance period. This underscores the necessity of strengthening public health systems to deliver quality maternal and child healthcare, alongside integrating CH with health services through referral systems or vouchers and addressing structural barriers like transportation. Furthermore, the persistent prevalence of emotional stress, trauma, and domestic abuse highlights that financial interventions alone cannot fully mitigate psychosocial and mental health needs, necessitating targeted, trauma-informed services and gender-sensitive social protection programs.

Building long-term resilience requires a multi-pronged approach that extends beyond immediate cash provision. We recommend improving cash distribution to ensure timely and consistent disbursements for long-term health planning and adopting a tailored “cash plus” model with targeted support for high-cost obstetric care or vulnerable groups. Crucially, investing in health and financial literacy through community-based education will empower recipients to optimize spending and enhance health-seeking behaviors, while linking families to micro-savings or microinsurance schemes can foster greater financial resilience. Finally, cross-sector collaboration aligning CH programs with national health priorities and fostering coordination among humanitarian, government, and development actors is essential to strengthen health system capacity and ensure equity by continually monitoring CH access and outcomes for sustained impact beyond the intervention period.

### 4.5. Limitations

Our study, despite its comprehensive assessment of CH’s financial and healthcare impacts, had several limitations. Since the age of participants was statistically different between baseline and endline, there are concerns regarding non-compatibility; however, both groups were in the same age range of 25–35 years. Inconsistent data collection, especially for income, constrained comparative and associative analyses, and some variables lacked sufficient variability for robust statistical assessment. We used a non-randomized sample, which may limit the generalizability of the findings. For the multivariable regression analysis only, “Healthcare Expenditure (USD)” served as the dependent outcome variable, while related Access and Preference indicators were utilized as independent predictor variables (covariates) to explore the determinants of the observed change in expenditure, since sample size constraints prevented us from conducting additional stratified regression analyses for the other outcome variables related to access and financial burden. Additionally, the sample size, primarily for quality assurance, also precluded in-depth or stratified analyses. Furthermore, reliance on self-reported preferences introduced potential framing bias, and despite rapid endline data collection, recall bias remained a possibility. Nevertheless, the integration of qualitative and quantitative data through triangulation enhanced the validity and credibility of our findings.

## 5. Conclusions

This study demonstrates that CH assistance can significantly enhance healthcare access and reduces financial burdens for highly vulnerable pregnant women and their families in climate-affected populations, leading to improved health-seeking behaviors and physical well-being immediately post-emergency. While CH proved instrumental in improving financial resilience and even addressing critical issues like food security and domestic violence, it also highlighted persistent challenges, including an equity gap in accessing comprehensive care, the nuanced impact of financial constraints, and the pervasive, unmet psychosocial needs in disaster contexts. Therefore, for sustainable impact, future interventions must move beyond singular cash injections to adopt integrated “cash plus” models. These should combine transparent, consistent financial aid with strengthened public health systems, targeted psychosocial support, and robust investments in health and financial literacy, all underpinned by cross-sector collaboration and continuous monitoring for equitable, long-term resilience. This comprehensive approach is essential to truly transform maternal and child health outcomes in crisis-affected, resource-constrained settings.

## Figures and Tables

**Table 1:** Distribution of demographic, healthcare expense, and access-related characteristics.

	Baseline (n = 1000)	Endline (n = 785)	Difference (95% CI)	*p*-Value
	**Mean (95% CI)**			
**Age (years)**	31 (30.5, 31.3)	28 (27.49, 28.31)	2.6 (1.7, 3.5)	**<0.001**
Household income (USD/month) *	54 (53.5, 55.9)	-	-	-
**Healthcare expenditure (USD)**	9.3 (8.2, 10.5)	25 (21.3, 28.5)	−16.1 (−25.8, −6.4)	**0.01**
	**Percentage**			
**Number of children**				
No children < 2 years old	23.3%	16.6%	0.1 (0.05, 0.2)	**<0.001**
1 child < 2 years old	74.9%	81.1%	−0.1 (−0.2, −0.05)	**<0.001**
More than 1 child < 2 years old	1.8%	2.3%	−0.01 (−0.02, 0.02)	0.6
**Level of education**				
No formal education	87%	87%	0.001 (−0.05, 0.05)	0.9
Primary education	10%	9%	0.03 (−0.01, 0.08)	0.1
Secondary education	2%	3%	−0.03 (−0.05, −0.01)	**0.03**
Higher level than secondary	1%	1%	−0.01 (−0.02, 0.001)	0.2
Primary sources of household income *				
Daily wage	81.8%	-	-	-
Monthly income	16.5%	-	-	-
**Barriers to accessing health services**				
Availability of services	25%	46%	−0.2 (−0.3, −0.1)	**<0.001**
Financial	55%	70%	−0.1 (−0.3, −0.1)	**<0.001**
Transportation	46%	73%	−0.3 (−0.3, −0.2)	**<0.001**
Forgone medical care in the past due to treatment costs	68%	97%	−0.3 (−0.3, −0.2)	**<0.001**
Percentage of CH assistance allocated to healthcare services **	-	22%	-	-
Number of cash installments received by the families of pregnant women **				
Once only	-	4%	-	-
Twice	-	31%	-	-
Thrice	-	8%	-	-
Four times	-	47%	-	-
Five times	-	10%	-	-
**Overall access to healthcare services**	58%	98%	−0.46 (−0.52, −0.39)	**<0.001**
Accessing emergency services	71%	30%	0.38 (0.31, 0.45)	**<0.001**
Accessing regular physicians	20%	69%	−0.45 (−0.52, −0.38)	**<0.001**
Accessing government facilities	43%	58%	−0.13 (−0.2, −0.05)	**<0.001**
Accessing private facilities	10%	41%	−0.31 (−0.36, −0.25)	**<0.001**
**Preference for healthcare spending**				
Medication	30%	95%	−0.65 (−0.71, −0.58)	**<0.001**
Consultations	6%	71%	−0.65 (−0.69, −0.61)	**<0.001**
Treatment	10%	44%	−0.34 (−0.39, −0.29)	**<0.001**
Laboratory testing	5%	40%	−0.35 (−0.4, −0.31)	**<0.001**
Labor and delivery	0.4%	0.4%	0.0004 (−0.009, 0.009)	0.9
**Choice between healthcare and other needs**				
Food	54%	99%	−0.4 (−0.5, −0.38)	**<0.001**
Livestock	1%	4%	−0.03 (−0.04, −0.01)	**0.03**
Rent	3%	3%	−0.03 (−0.04, −0.05)	**0.006**
**Access to meals**				
Access to nutritious meals	21%	97%	−0.7 (−0.8, −0.7)	**<0.001**
Two meals per day	74%	26%	0.5 (0.4, 0.5)	**<0.001**
Three meals per day	19%	74%	−0.5 (−0.6, 0.4)	**<0.001**
**Household coping strategies**				
Borrowing money	61%	92%	−0.31 (−0.38, −0.25)	**<0.001**
Support from the family	5%	41%	−0.37 (−0.4, −0.32)	**<0.001**
Reducing spending on other needs	21%	31%	0.01 (−0.01, 0.02)	0.2
Selling livestock	1.8%	0.9%	−0.01 (−0.02, 0.01)	0.14
Selling other assets	0.5%	1%	−0.5 (−0.07, −0.04)	**<0.001**
Skipping meals	0%	5%	−0.05 (−0.12, 0.1)	0.1

* Only measured at baseline; ** Only measured at endline; Bold is for those which are statistically significant.

**Table 2:** Associations between healthcare expenditure and access and preferences for healthcare services at baseline and endline.

	Baseline β (95% CI)	*p*-Value	Endline β (95% CI)	*p*-Value
**Age**	−21.8 (−71.2, 27.6)	0.4	104.4 (−59.2, 268.1)	0.2
**No education**	21.1 (−920, 963)	0.9	−4120 (−6795.9, −1444)	**0.003**
**One child under 2 years**	−135 (−928, 659)	0.7	1744 (−859, 4347)	0.189
**No child under 2 years**	2253 (−637, 5135)	0.1	−1022 (−7513, 5468)	0.7
**Access to healthcare**	513 (−794, 1821)	0.4	-	**-**
Accessing emergency services	−191 (−2032, 1649)	0.8	−976 (−3240, 1287)	0.4
Accessing regular physicians	382 (−1549, 2313)	0.7	-	**-**
Accessing government facilities	−596 (−1989, 797)	0.4	−4568 (−6717, −2419)	**<0.01**
Accessing private facilities	−331 (−2021, 1358)	0.7	-	**-**
**Preference for healthcare spending**				
Medication	−283 (−1068, 501)	0.5	855 (−3424, 5135)	0.7
Consultations	1454 (324.5, 2584)	**0.01**	3595 (1451, 5738)	**0.001**
Treatment	−363 (−1376, 649)	0.5	3066 (919, 5212)	**0.005**
Laboratory testing	420 (−980, 1820)	0.5	3100 (764, 5438)	**0.009**
Labor and Delivery	−1009 (−6577, 4558)	0.7	7692 (4669, 10,716)	**<0.001**

Note: Health care expenditure (continuous variable) was the outcome variable; *p*-value < 0.05 was significant; Bold is for those which are statistically significant. The expenditure was in PKR and was analyzed as such. Conversion to USD was performed for comparative reporting using historical currency exchange data for the time when the intervention took place. Conversion available at: https://www.oanda.com/currency-converter/en/?from=PKR&to=USD&amount=3595 (accessed on 8 September 2025).

## Data Availability

The data presented in this study are not publicly available due to the privacy and confidentiality of the study participants. However, the data supporting the findings of this article are available upon reasonable request from the corresponding author, provided that the request complies with ethical guidelines and institutional data sharing policies.

## Acronyms

CVA	Cash and Voucher Assistance
CI	Confidence Interval
CRC	Canadian Red Cross
MUAC	Mid-Upper Arm Circumference
CH	Cash for Health
NorCross	Norwegian Red Cross
PKR	Pakistani Rupee
PRCS	Pakistan Red Crescent Society
QA	Quality Assurance
STATA	Statistical software package used for data analysis
UNFPA	United Nations Population Fund
USD	United States Dollar
